# Lifestyle Triple P: a parenting intervention for childhood obesity

**DOI:** 10.1186/1471-2458-12-267

**Published:** 2012-04-03

**Authors:** Sanne MPL Gerards, Pieter C Dagnelie, Maria WJ Jansen, Lidy OHM van der Goot, Nanne K de Vries, Matthew R Sanders, Stef PJ Kremers

**Affiliations:** 1 Department of Health Promotion, and Nutrition and Toxicology Research Institute Maastricht (NUTRIM), Maastricht University, Maastricht, The Netherlands; 2 Department of Epidemiology, Maastricht University, Maastricht, The Netherlands; 3 School of Public Health and Primary Care (Caphri), Maastricht University, Maastricht, The Netherlands; 4 South Limburg Public Health Services, Geleen, The Netherlands; 5 Department of Youth Health Care, Public Health Services, Geleen, The Netherlands; 6 Parenting and Family Support Centre, School of Psychology, The University of Queensland, Brisbane, Australia

## Abstract

**Background:**

Reversing the obesity epidemic requires the development and evaluation of childhood obesity intervention programs. Lifestyle Triple P is a parent-focused group program that addresses three topics: nutrition, physical activity, and positive parenting. Australian research has established the efficacy of Lifestyle Triple P, which aims to prevent excessive weight gain in overweight and obese children. The aim of the current randomized controlled trial is to assess the effectiveness of the Lifestyle Triple P intervention when applied to Dutch parents of overweight and obese children aged 4–8 years. This effectiveness study is called GO4fit.

**Methods/Design:**

Parents of overweight and obese children are being randomized to either the intervention or the control group. Those assigned to the intervention condition receive the 14-week Lifestyle Triple P intervention, in which they learn a range of nutritional, physical activity and positive parenting strategies. Parents in the control group receive two brochures, web-based tailored advice, and suggestions for exercises to increase active playing at home. Measurements are taken at baseline, directly after the intervention, and at one year follow-up. Primary outcome measure is the children’s body composition, operationalized as BMI z-score, waist circumference, and fat mass (biceps and triceps skinfolds). Secondary outcome measures are children’s dietary behavior and physical activity level, parenting practices, parental feeding style, parenting style, parental self-efficacy, and body composition of family members (parents and siblings).

**Discussion:**

Our intervention is characterized by a focus on changing general parenting styles, in addition to focusing on changing specific parenting practices, as obesity interventions typically do. Strengths of the current study are the randomized design, the long-term follow-up, and the broad range of both self-reported and objectively measured outcomes.

**Trial Registration:**

Current Controlled Trials NTR 2555

**MEC AzM/UM:**

NL 31988.068.10 / MEC 10-3-052

## Background

Overweight and obesity are having increasing public health impact worldwide. In 2003, between 12% and 19% of 4- to 8-year-old children in the Netherlands were overweight, and about 2 to 4% were obese [1]. More recent data (2007 – 2010) show that 17% of the 7- to 8-year-old children are overweight and 5% are obese [2]. Overweight children are at increased risk of becoming obese adults [3] and of developing cardiovascular diseases or type 2 diabetes [4]. Moreover, these children often suffer from social consequences like teasing and discrimination [5] which may influence their mental health. It is therefore important to develop and evaluate interventions to reverse this trend of increasing prevalence. In recent decades, childhood obesity interventions have been developed and evaluated, with some promising results [6-8], but there is still a lack of sound theory- and evidence-based interventions to prevent overweight in children [9].

There are several reasons for focusing efforts to prevent excessive weight gain on children at a relatively young age. First, overweight at a young age predicts excessive weight gain in the future [10]. Also, children have a relatively short history of unhealthy habits, which may make it easier to change these behaviors compared to adult populations. Finally, prevention of excessive weight gain in children is based on a different mechanism than prevention of weight gain in adults: children can reduce their BMI while growing without losing weight, whereas adults have to lose weight in order to reduce their BMI [11].

The role of the parents in the development of children’s weight status is increasingly emphasized in intervention studies on the treatment and prevention of childhood obesity [8,12]. Parents are the primary caregivers, who are largely responsible for their children’s nutrition and physical activity patterns, particularly in the early years of life. It is therefore important to target both general parenting styles and parenting practices. Unlike parenting practices, which focus on specific parenting behaviors relating to aspects like dietary behavior and physical activity, parenting styles refer to parent–child interactions across a wide range of situations. Parenting styles are regarded as the context in which behavior-specific parenting takes place [13,14]. A recent review indicated that interventions aimed at changing parenting styles are effective in the prevention and management of childhood obesity [15]. In addition, parenting interventions seem to have positive long-term effects on a range of other youth outcomes, viz. mental, emotional and behavioral disorders, as well as on successful developmental competence [16].

The Triple P Positive Parenting Program is a multi-level parenting and family support strategy developed by the University of Queensland in Brisbane, Australia [17]. Triple P is based on social learning principles and adopts a system-contextual or ecological perspective in supporting parents. The program is internationally supported and several derivative programs have been developed to address parents’ special needs. One of these derivative programs is Lifestyle Triple P [18]. It is a parent-focused group program that addresses three topics: nutrition, physical activity, and positive parenting. The efficacy of Lifestyle Triple P has been tested in a randomized controlled trial (RCT) in Australia [19]. West and colleagues showed that their intervention significantly decreased children’s body size and body fat, decreased their weight-related problem behavior, increased parental confidence in managing weight-related problem behavior, and decreased ineffective parenting [19,20]. The intervention has not been tested outside Australia. In addition, a limitation noted by the authors of the Australian study was that their measures of lifestyle patterns had low reliability and sensitivity [20], and that they had not measured the effects of the intervention on other family members (parents or siblings). Based on the process evaluation of the Australian study, in which parents indicated that the program was too short, the original 12-session Lifestyle Triple P program was extended to 14 sessions. Before implementation of the intervention on a national scale in the Netherlands, we decided to assess the effects of the adapted version of Lifestyle Triple P in the Dutch context.

The aim of the current RCT is to assess the effectiveness of the Lifestyle Triple P intervention in the Netherlands in addressing overweight and obese children aged 4–8 years. The study has been named GO4fit, and aims to assess changes in children’s anthropometric outcomes, anthropometric outcomes of other family members (parents and siblings), parenting practices, parental feeding style, parenting style, parental self-efficacy and children’s energy balance-related behaviors.

## Methods/design

### Study design

**Figure 1:**
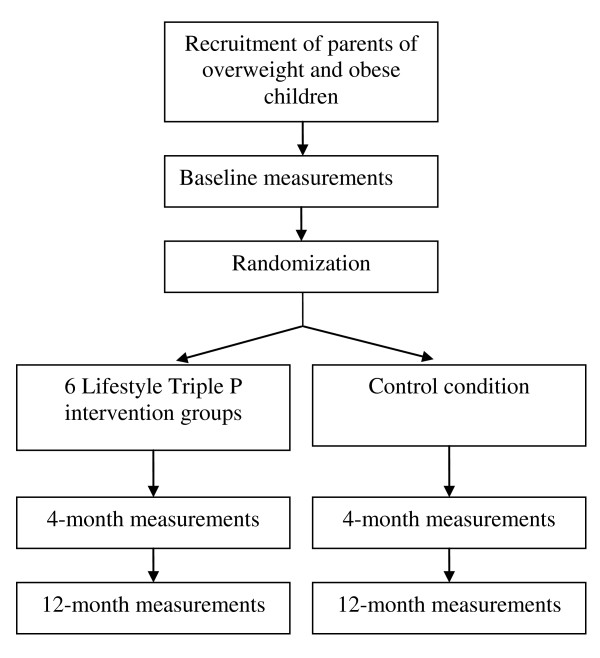
Study design.

The effectiveness of the Lifestyle Triple P intervention is being tested in the southern part of the Dutch province of Limburg, using an RCT design. After baseline measurements, parents of overweight children are randomly allocated to either the intervention group or the control group. The intervention group receives the Lifestyle Triple P intervention, whereas parents in the control group receive information on healthy nutrition, physical activity, and positive parenting. To evaluate the effect of the intervention, participants are measured again immediately following the intervention (4 months after baseline), and at 12 months after baseline (see Figure 1 for the study design). Baseline measurements and intervention groups are started as soon as enough participants per location have been recruited (i.e. a minimum of 10: 5 for the control group and 5 for the intervention group). The Medical Ethics Committee of the University Hospital Maastricht and Maastricht University approved the study protocol (reference number NL 31988.068.10 / MEC 10-3-052).

### Target population

The Southern Limburg regions has 608,885 inhabitants [21], 49.8% (303,225) of whom live in the municipalities of Maastricht, Sittard-Geleen and Heerlen. About 10% are aged 4 to 8 years. The estimated prevalences of overweight and obesity in the 4–8 year age group are 9.5% and 2.3% respectively, implying an eligible population of 3,578 children for the current study.

### Recruitment of the study sample

The participants are being recruited using four strategies: two strategies that use the Dutch Youth Health Care (YHC) system, one using mass media and one using personal letters. Health professionals working in the YHC system have been instructed to refer parents of overweight children to the Lifestyle Triple P intervention. In addition, parents of children who are overweight according to the YHC’s medical records have been approached for participation in the intervention. Finally, mass media materials (a brochure, a poster, advertisements in newspapers, and a website) have been developed to inform parents about the intervention and to offer them the opportunity to register for the intervention. We have also sent invitation letters to parents of primary school children. Recruitment of participants for the current RCT started in December 2010.

#### Participants

We aim to recruit a study population of 84 child-parents triads. Parents of children aged between 4 and 8 years (at inclusion) are eligible for participation if their child is considered overweight or obese, based on the BMI, using the international sex- and age-specific cut-off points proposed by Cole et al [22]. Parents who agree to participate in the study and both sign the informed consent form are included in the current study.

#### Power calculation

The sample size calculation was based on the difference between the intervention condition and the control condition in terms of BMI points. A difference of 0.30 BMI points between the intervention and control conditions was expected to be relevant, based on previous studies, e.g. Robinson 1999 [23]. Based on an alpha value of 0.05 and a power of 0.90, adjusting for attrition and nesting effects within the groups, we aimed to randomly assign 84 families to the 6 Lifestyle Triple P groups (intervention) or to the control condition.

### Randomization

The randomization scheme has been generated by an independent researcher (PCD), who is not directly involved in the data collection or intervention delivery, using a block size of four and sealed envelopes. The randomization is concealed to all other members of the study team. After baseline measurements, participants are randomly allocated to either the intervention or the control condition. A member of the study team (SMPLG), who is blinded to the randomization scheme, calls the research institute by telephone in the presence of the parents to enquire for their group allocation, and then informs the parents.

### Lifestyle Triple P Intervention

#### Objectives

Key objectives of the Lifestyle Triple P intervention are:

improving children’s dietary intake, activity levels, and weight status;

increasing parenting skills and confidence in managing children’s weight-related behavior;

reducing parents’ use of coercive and permissive discipline practices to their children;

improving parents’ communication about health and nutrition;

reducing parenting stress associated with raising healthy children.

#### Structure

The participants who are assigned to the intervention condition receive a 14-week Lifestyle Triple P intervention which consists of eight weekly 90-minute parental group sessions, followed by two weekly 15–30 minute telephone sessions, one further 90-minute group session, two weekly 15–30 minute telephone sessions, and a final 90-minute group session. The groups are composed of either one or both parents of at most 9 families, and each group is led by two Lifestyle Triple P facilitators. These are health professionals who have been accredited after attending an official 3-day Triple P training course and an additional Lifestyle Triple P training day. The group sessions take place at three different buildings of the regional Public Health Services (at Heerlen, Geleen, and Maastricht). To ensure intervention fidelity (defined as the extent to which the intervention is implemented as intended [24]), the facilitators meet on a regular basis with a member of the study team (SMPLG) for supervision. Participation in the intervention is free of charge. Each family is provided with a parent workbook, a recipe book, and an active games booklet to support the information presented in the sessions. All the materials have been translated into Dutch for the current trial, according to the following process. First, translators of Triple P International translated the materials from English to Dutch. Hereafter, all translations were reviewed by a native Dutch speaker (SMPLG). The translation and reviewing process were conducted in line with already existing Dutch Triple P materials. A pilot study was undertaken to test specific parts of the Lifestyle Triple P intervention for feasibility and acceptability.

#### Content

Lifestyle Triple is a family intervention strategy to prevent and treat childhood obesity. The intervention consists of active skills training methods based on self-regulation principles, to provide parents with new knowledge and skills. In the first sessions, parents individually formulate realistic goals for change, with the help of the trainer and based on their child’s dietary intake and activity levels. Parents are instructed on a range of nutrition strategies (e.g., establishing eating routines and providing healthy foods), physical activity strategies (e.g., providing active games and playing with your child), and positive parenting strategies (e.g., spending quality time with your child and giving frequent praise). An overview of the intervention topics is shown in Table 1. The telephone sessions aim to provide parents with individual support in the implementation of strategies at home.

**Table 1:** Overview of the topics and strategies in each Lifestyle Triple P session

**Session**	**Topics**	**Strategies**
Session 1: Preparing for change	Nature and causes of obesity Overview of Lifestyle Triple P Readiness to change	
Session 2: Understanding nutrition	Increasing children’s self-esteem Food groups and daily servings Nutrition goals	Spending quality time with your child Talking to your child Showing affection
Session 3: Understanding physical activity	Encouraging healthy behaviors Physical activity goals Increasing incidental activity Reducing sugar intake	Giving frequent praise Setting a good example Replacing foods high in added sugar Providing water as a regular drink Spending family leisure time in active ways Encouraging active means of transport
Session 4: Using rewards and modifying recipes	Using behavior charts Reducing fat intake Modifying recipes	Using behavior charts Buying low-fat foods Using low-fat cooking methods Replacing high-fat ingredients
Session 5: Limiting sedentary activity and reading food labels	Limiting sedentary activities Establishing ground rules Reading food labels	Establishing clear ground rules Using targeted discussion to deal with rule-breaking Reading food labels Limiting sedentary activities
Session 6: Playing active games	Providing active alternatives Improving movement skills	Providing active games Playing with your child
Session 7: Providing healthy meals	Establishing eating routines Providing healthy meals/snacks Children’s participation in sport	Establishing eating routines Providing healthy meals and snacks Encouraging participation in sport
Session 8: Managing problem behavior	Managing problem behaviors	Using planned ignoring for minor misbehavior Giving clear, calm instructions Backing up instructions with consequences, quiet time, or time-out
Session 9: Using Lifestyle Triple P strategies 1	Implementing strategies	
Session 10: Using Lifestyle Triple P strategies 2	Implementing strategies	
Session 11: Planning ahead	Family survival tips High-risk situations Planned activities routine	
Session 12: Using planned activities 1	Implementing planned activities routine	
Session 13: Using planned activities 2	Implementing planned activities routine	
Session 14: Program Close	Progress review Maintaining changes Problem solving for the future	

### Control condition

Participants who are randomized to the control condition receive two brochures (one on healthy nutrition and physical activity, and one on positive parenting), web-based tailored advice on setting a good example to their child, and suggestions for exercises to increase active play at home.

### Outcome measures

Outcome measures are assessed at baseline (one or two weeks before the start of the intervention), at 4 months (immediately after the intervention), and at one year follow-up. Primary outcome measure is children’s body composition, operationalized as BMI z-score, waist circumference, and fat mass. Secondary outcome measures are children’s dietary behavior and physical activity level, parenting practices, parental feeding style, parenting styles, parental self-efficacy, and body composition of family members (parents and siblings).

#### Anthropometry

Anthropometric measurements in the children are conducted during a visit to the Public Health Service office. If one or both parents are willing to participate, they are measured as well. In addition, if siblings (if applicable) are present at the visit and are willing to participate, they are also measured. Measurements are performed by a YHC professional who is blinded for group allocation, using a standardized protocol. The participating YHC professional has many years of experience in performing body measurements and has attended a one-day training course to perform the measurements in accordance with the protocol.

Weight is measured using an electronic portable scale (standardized Seca 899) to the nearest 0.1 kg, with the subject wearing only underwear. Height is measured using a portable stadiometer (Seca 214) with an accuracy of 1 mm. Both measurements are used to calculate BMI and BMI z-scores. Waist circumference is measured with a flexible tape to the nearest 1 mm. Biceps and triceps skinfold thickness is measured to the nearest 0.1 mm using a Harpenden skinfold caliper. Each skinfold is measured three times and the median is used. If two of the scores differ by more than 10%, another three skinfolds are measured, and the median of the six values is used.

#### Accelerometry

Children’s physical activity levels are measured using an Actigraph accelerometer (Actigraph, Pensacola, Florida). Children are asked to wear the accelerometer for seven consecutive days, preferably in the week after the anthropometric measurements. The epoch (time frame) is set at 15 sec.

#### Questionnaire

Parents are asked to fill out a questionnaire at baseline, at posttest and at one year follow-up. The questionnaire has been compiled from validated scales, and assesses the following variables:

Demographics: gender, age, household and family composition, educational level of the parents, ethnicity of the child and the parents, and work situation of the parents;

Energy balance-related behaviors of the child: screen-viewing behavior, snacking behavior, soft-drink consumption, fruit and vegetable consumption and physical activity level [25];

Parenting practices: monitoring, restriction, pressure to eat, and perceived responsibility [26];

Parental feeding style: instrumental feeding, emotional feeding, control, and encouragement [27];

Parenting style: restrictiveness and nurturance [28] and psychological control [29];

Parenting self-efficacy: satisfaction about one’s own efficacy and effectiveness at solving problems [30];

Personality characteristics of the child: surgency/extraversion, negative affectivity, and effortful control [31].

Parental satisfaction with the intervention is measured by including process evaluation questions in the post-test questionnaire at 4 months, and questions about changes in problem behavior and management of problem behavior in the 1-year questionnaire.

### Statistical analysis

Descriptive statistics will be used to describe subject characteristics, including baseline values of primary and secondary outcome measures. Continuous variables will be presented as means and standard deviations. Categorical data will be presented as percentages of respondents within each of the possible categories.

Both univariate and multivariate multilevel analyses (to control for potential nesting effects within the Lifestyle Triple P groups) will be conducted to determine the effect of the intervention on changes in children’s BMI z-score, children’s waist circumference, children’s fat mass, children’s lifestyle (screen-viewing behavior, snacking behavior, soft-drink consumption, fruit and vegetable consumption, and physical activity level), parenting self-efficacy, parental skills, and the BMI, waist circumference, and fat mass of both the parents and the siblings. All analyses will be performed according to intention-to-treat analyses, and additional per protocol analyses will be performed. Models will be adjusted for relevant confounders such as children’s age, gender, and ethnicity, and parental socio-economic status.

## Discussion

The purpose of this paper was to describe the design of the GO4fit study, which is currently testing the effectiveness of the Lifestyle Triple P intervention in the Netherlands.

Strengths of the current study include its RCT design, the long-term follow-up, and the broad range of both self-reported and objectively assessed outcome measures. Challenges of the study are mainly related to recruitment issues and intervention implementation. Recruitment issues are due to the difficulty of fitting the intervention into the Dutch Youth Health Care system, parents’ underestimation of their child’s weight status [32], and low recognition among parents of the relevance of the intervention. Intervention implementation issues are related to high quality intervention delivery and intervention fidelity.

In an effort to involve important stakeholders in the recruitment of participants and the implementation of the Lifestyle Triple P intervention, we have secured the support of the Academic Collaborative Centre for Public Health in Limburg. The Academic Collaborative Centre represents a collaboration between policy (municipal authorities), practice (Public Health Service) and research (Maastricht University) with the aim of improving public health [33]. We have tried to further optimize the implementation of the Lifestyle Triple P intervention in the Netherlands (e.g., translation of the intervention materials, training of the Lifestyle Triple P facilitators) by collaborating with the Family Support Centre of the University of Queensland, Brisbane, Australia.

If the intervention proves effective, implementation studies will be needed, including research on ways of reaching low-SES groups and ways of integrating the program in existing YHC structures. A notable characteristic of the intervention is its focus on changing general parenting styles, in addition to solely focusing on changing specific parenting practices, as obesity interventions typically do [34]. We expect that interventions focusing on changing general parenting styles will have a large and sustained impact on the children’s energy balance-related behavior. In addition, we expect these interventions to have an impact on a broad range of specific other parenting practices relating to multiple child outcomes [34], indicating a potentially large public health impact [15]. The first results of the RCT are expected end 2013.

## Abbreviations

BMI: Body mass index; RCT: Randomized controlled trial; SES: Socio-economic status; YHC: Youth health care.

## Competing interests

All authors declare that they have no competing interests.

## Authors’ contributions

SMPLG was the primary investigator in this study and wrote the first draft of the paper. PCD, MWJJ, LOHMvdG, NdV, MRS and SPJK were involved in revising the manuscript. All authors have read and approved the final manuscript.

## Pre-publication history

The pre-publication history for this paper can be accessed here:

http://www.biomedcentral.com/1471-2458/12/267/prepub
